# Adoptive T‐cell therapy targeting Epstein–Barr virus as a treatment for multiple sclerosis

**DOI:** 10.1002/cti2.1444

**Published:** 2023-03-21

**Authors:** Corey Smith, Rajiv Khanna

**Affiliations:** ^1^ QIMR Berghofer Centre for Immunotherapy and Vaccine Development, Infection and Inflammation Program QIMR Berghofer Medical Research Institute Herston QLD Australia

**Keywords:** Epstein–Barr virus, immunotherapy, multiple sclerosis, T cells

## Abstract

Emergence of a definitive link between Epstein–Barr virus (EBV) and multiple sclerosis has provided an impetus to develop immune‐based therapies to target EBV‐infected B cells. Initial studies with autologous EBV‐specific T‐cell therapy demonstrated that this therapy is safe with minimal side effects and more importantly multiple patients showed both symptomatic and objective neurological improvements including improved quality of life, reduction of fatigue and reduced intrathecal IgG production. These observations have been successfully extended to an ‘off‐the‐shelf’ allogeneic EBV‐specific T‐cell therapy manufactured using peripheral blood lymphocytes of healthy seropositive individuals. This adoptive immunotherapy has also been shown to be safe with encouraging clinical responses. Allogeneic EBV T‐cell therapy overcomes some of the limitations of autologous therapy and can be rapidly delivered to patients with improved therapeutic potential.

## Graphical abstract

Adoptive immunotherapy targeting Epstein–Barr virus has emerged as a potential therapeutic strategy for the treatment of multiple sclerosis. We have developed a novel manufacturing process, which can offer these T‐cell therapies as an off‐the‐shelf treatment.

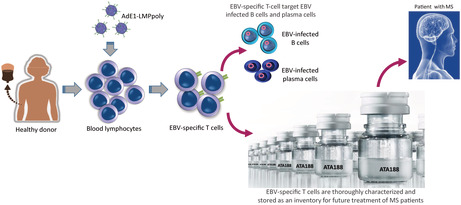

## Introduction

**Figure 1:**
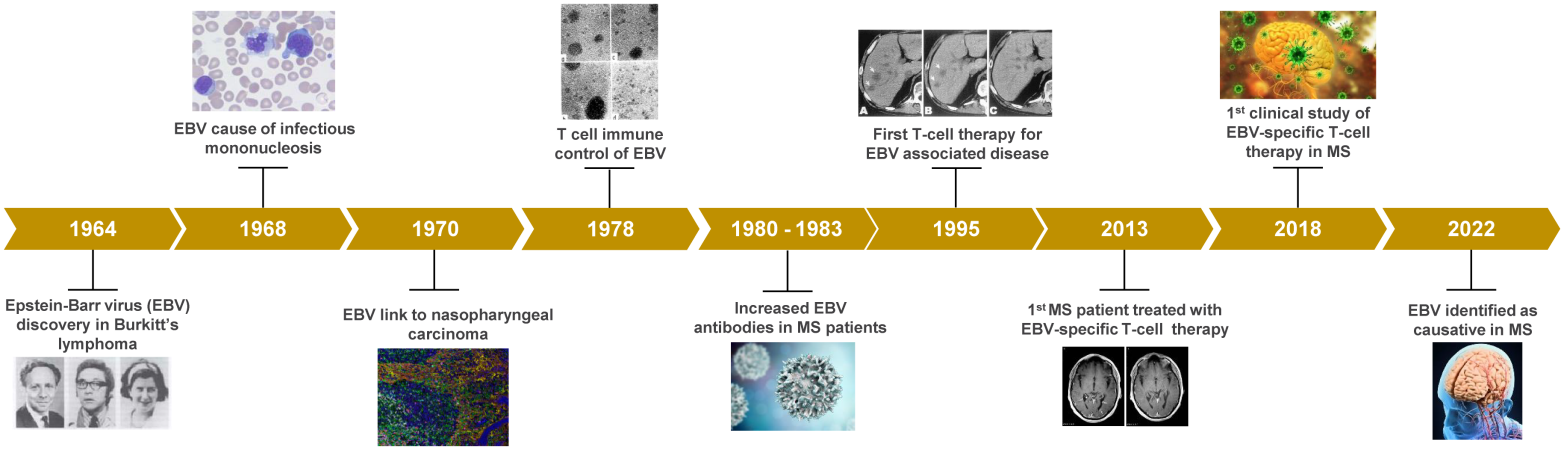
Timeline showing the major breakthroughs in Epstein–Barr virus (EBV) research leading to the establishment of links with various diseases including multiple sclerosis (MS) and the development of EBV‐specific T‐cell therapy for EBV‐associated diseases. This timeline is based on previously published studies.^1^, ^2^, ^8^, ^9^, ^10^, ^12^, ^13^, ^47^

Accumulating evidence over the last two decades has provided increasing insight into the causative role of Epstein–Barr virus (EBV) in the development of multiple sclerosis (MS).^1^, ^2^ EBV is a gammaherpesvirus that infects > 90% of the worldwide population and has been linked to an increasing number of malignant and nonmalignant diseases since its discovery in 1964 (Figure 1).^3^, ^4^, ^5^, ^6^ It has a unique lifecycle attributable to its ability to induce the lifelong transformation of B cells following primary infection. Dissemination of the viral genome in the host during latency can occur via replication of the viral episome alone.^7^ As a consequence, viral persistence occurs within the host without the need for the replication of the whole virus. However, periodic reactivation does occur, including in healthy immunocompetent individuals, which allows viral shedding and infection of a new host. This persistence of EBV in the host is also predicated on its ability to evade clearance by the host immune system. While normally in equilibrium with the host immune system, leading to stable lifelong asymptomatic infection in most people, it is disequilibrium in host immune control of EBV‐transformed cells that plays an important role in most EBV‐associated diseases. It has been speculated that poor immune control of EBV‐transformed cells contributes to the pathogenesis of MS and that mechanisms that restore efficient immune control could provide an approach to control the severity of disease in MS patients. Over the past decade, our research group has been developing an immunotherapeutic approach designed to harness EBV‐specific T cells to target the antigenic determinants that are likely critical for the maintenance of EBV‐infected B cells in MS patients.^8^, ^9^ This strategy is based on the wealth of knowledge gained over the last few decades into the latent lifecycle of EBV and its interaction with the human immune system.

## EBV latency and immune surveillance

Latent infection of B cells with EBV is characterised by the coordinated expression of latent genes with B‐cell differentiation status.^10^, ^11^ EBV infection of naïve B cells is followed by the coordinated differentiation of B cells through a proliferative state, a germinal centre phenotype and a memory phenotype. The proliferative state expresses a latency III profile, akin to the profile of *in vitro* derived EBV‐transformed lymphoblastoid cells lines, which includes the LMP 1 and 2, and the EBV nuclear antigens (EBNA) 1–6. The germinal centre phenotype has a restricted latency II pattern characterised by the expression of EBNA1, LMP1 and LMP2. The memory phenotype corresponds to the latency I/0 state whereby gene expression is limited to EBNA1 and the transformed cells are quiescent. These latency patterns also define the EBV gene expression patterns present in EBV‐associated malignancies. The latency III profile is associated with lymphoproliferative diseases that arise in immunocompromised patients, whereas the latency I and II profiles are present in cancers that arise in patients that are otherwise immunocompetent.^10^, ^12^, ^13^ These observations demonstrate that immune pressure plays a key role in regulating the survival of EBV‐transformed cells.^14^, ^15^

During primary lytic stages of infection, EBV uses many distinct proteins to prevent its clearance by the immune system.^16^ These proteins primarily act through the suppression of antigen processing and presentation via the MHC pathways and the regulation of cytokine expression, thus restricting T‐cell‐mediated clearance of lytically infected cells. However, for the majority of its lifecycle, EBV is within the latent state of infection and these immune evasion mechanisms do not function during latency. The highly proliferative latency III profile EBV‐transformed B cells are highly immunogenic and are rapidly surveilled by EBV‐specific T cells. Immune recognition is dominated by T cells specific for the immunodominant EBV nuclear antigens EBNA 3, 4 and 6.^17^, ^18^ As a consequence, the full latency III pattern of EBV gene expression is rarely detected in EBV‐associated diseases. Significant immune deficiency, such as in patients with acquired immunodeficiency syndrome or following organ transplantation, is usually required for the uncontrolled outgrowth of EBV‐transformed B cells with a latency III profile. The most prevalent diseases associated with latency III are the post‐transplant lymphoproliferative diseases (PTLD).^19^ In contrast to the highly immunogenic latency III, cells displaying a latency I or II profile are much less immunogenic. The proteins associated with latency I & II, EBNA1 and LMP 1 and 2 all show evidence of reduced immune recognition.^20^

EBNA1 plays a critical role in maintenance of the EBV genome in latently transformed cells by tethering the EBV episome to the host genome and binding to OriP, initiating replication of the EBV genome.^21^, ^22^ This allows spread of the viral genome into daughter cells without the need for lytic replication. EBNA1 is therefore critical in all stages of latency. The glycine–alanine repeat (GAr) sequence within EBNA1 plays a critical role in regulating its cis‐translation and endogenous presentation of EBNA1 epitopes to CD8^+^ T cells.^23^, ^24^, ^25^, ^26^ Initially shown to restrict protein degradation by the proteasomal pathway, more recent studies have demonstrated that a purine bias in the GAr sequence generates G‐quadruplexes in the mRNA transcripts of EBNA1 that hampers its translation, limiting the amount of EBNA1 protein in EBV‐transformed cells.^23^, ^26^, ^27^ Despite this very effective mechanism of immune evasion, EBNA1‐reactive T cells are still found in a large proportion of the population and have been shown to be effective in both preclinical and clinical studies in controlling EBV‐associated disease.^9^, ^28^, ^29^, ^30^

The LMP1 and LMP2 proteins also play critical roles in the transformation of B cells. LMP1 provides a constitutive signal in infected cells, analogous to that provided by CD40 in activated lymphocytes.^31^ This drives nuclear factor kB signalling that is critical for cell transformation.^32^, ^33^ LMP1‐signalling is mediated through the formation of large protein aggregates in the cellular membranes.^34^ Unlike EBNA1, LMP1 does not appear to self‐regulate its own expression, rather these large aggregates are rapidly degraded by the autophagy pathway and are not efficiently processed for presentation via the MHC class I pathway.^35^, ^36^, ^37^ Interestingly, LMP1‐mediated nuclear factor kB signalling promotes the upregulation of MHC processing and presentation, rendering EBV‐infected cells highly susceptible to killing through the recognition of the EBNA3‐6 antigens.^38^ However, this does not lead to enhanced cis‐recognition of LMP1, which is not highly immunogenic and LMP1‐specific T‐cell responses are only detectable in a minority of healthy individuals.^36^ LMP2 comprises two protein LMP2A and LMP2B. It mimics B‐cell receptor signalling, a key step in the maturation and transformation of the infected cells.^39^ T‐cell immunity to LMP2 is more readily detected in healthy individuals.^17^, ^18^ However, studies have suggested that LMP2A can have an impact on antigen presentation to T cells and we have previously shown that LMP2‐specific T cells are less efficient at recognising EBV‐transformed B cells compared with EBNA3‐6‐specific T cells.^20^, ^40^ LMP‐specific T cells have also demonstrated efficacy in controlling EBV‐associated diseases.^41^, ^42^

As a consequence of these strategies to limit antigen recognition, T cells specific for EBNA1 and LMP1&2 are not only less abundant but they also appear to have altered maturation and reduced functionality compared with T cells specific for EBNA3‐6.^20^ Our observations have suggested that this can render EBNA1 and LMP1&2‐specific T cells more susceptible to immune evasion mechanisms mediated by EBV‐infected targets cells, which may further limit the clearance of cells with the latency I/II profile. However, we also noted that *in vitro* expansion and maturation of these T cells can overcome some of these immunosuppressive mechanisms.^20^

## EBV and disease pathogenesis in multiple sclerosis

Disease pathogenesis in MS is highly complex and involves both environmental and genetic factors, including infectious agents, vitamin D deficiency (because of lack of sun exposure) smoking, obesity and major histocompatibility antigens (HLA DRB1*1501).^43^, ^44^ The first suggestion of an infectious aetiology in MS was provided by Pierre Marie in 1894. Since then, both viral and bacterial pathogens have been promoted as infectious agents associated with MS.^45^, ^46^ Warner and Carp^47^ argued that late acquisition of EBV infection as a young adult or late adolescence may be a critical factor in the development of MS. They proposed that late acquisition of EBV leading to infectious mononucleosis may lead to a different type of pathogenesis when compared to early onset of EBV infection. Indeed, epidemiological studies have shown that early acquisition of EBV infection and a low rate of incidence of infectious mononucleosis in Asian, Polynesian and African population is coincident with lower incidence of MS.^48^ These early correlative studies have recently been further supported by one of the most definitive studies based on a cohort of 10 million US army personnel who were longitudinally followed for 20+ years and monitored for the development of MS and acquisition of viral infections, including EBV.^1^ This study demonstrated that EBV infection is a prerequisite for the development of MS and acquisition of EBV infection as a young adult increased the risk of MS diagnosis by 32‐fold. Previous studies by Serafini *et al*.^49^ provided the first evidence of EBV infection in MS lesions with co‐localisation of autoreactive T cells. They identified germinal centre‐like features in ectopic B‐cell follicles in MS brain lesions. They used *in situ* hybridisation for EBV‐encode small nuclear mRNA (EBER) to demonstrate the presence of EBV in B cells in the lesions. LMP1, EBNA2 and the lytic gene BFRF1 were also detected in the brains of MS patients.^50^, ^51^, ^52^ Subsequent studies reported the presence of EBNA1 and LMP2A in lesions. Hassani *et al*.^53^ confirmed the expression of EBER and EBNA1 and detected expression of the lytic gene BZLF1. More recently, Moreno *et al*.^54^ reported the presence of LMP1 in the majority of both MS and control brains, with higher LMP1 positivity associated with MS patients, and lesser expression of BZLF1. Gene expression profiling has also reported the expression of LMP1, LMP2A and EBNA3A in MS brain lesions.^55^ While far from definitive, and contested by other studies that did not find evidence for the expression of EBV proteins in MS patient brain tissue,^56^ these observations suggest that the association of EBV and MS may be attributable to an increased frequency of latently transformed B cells in the brain of MS patients and a failure of the host immune system to effectively control these cells.

**Figure 2:**
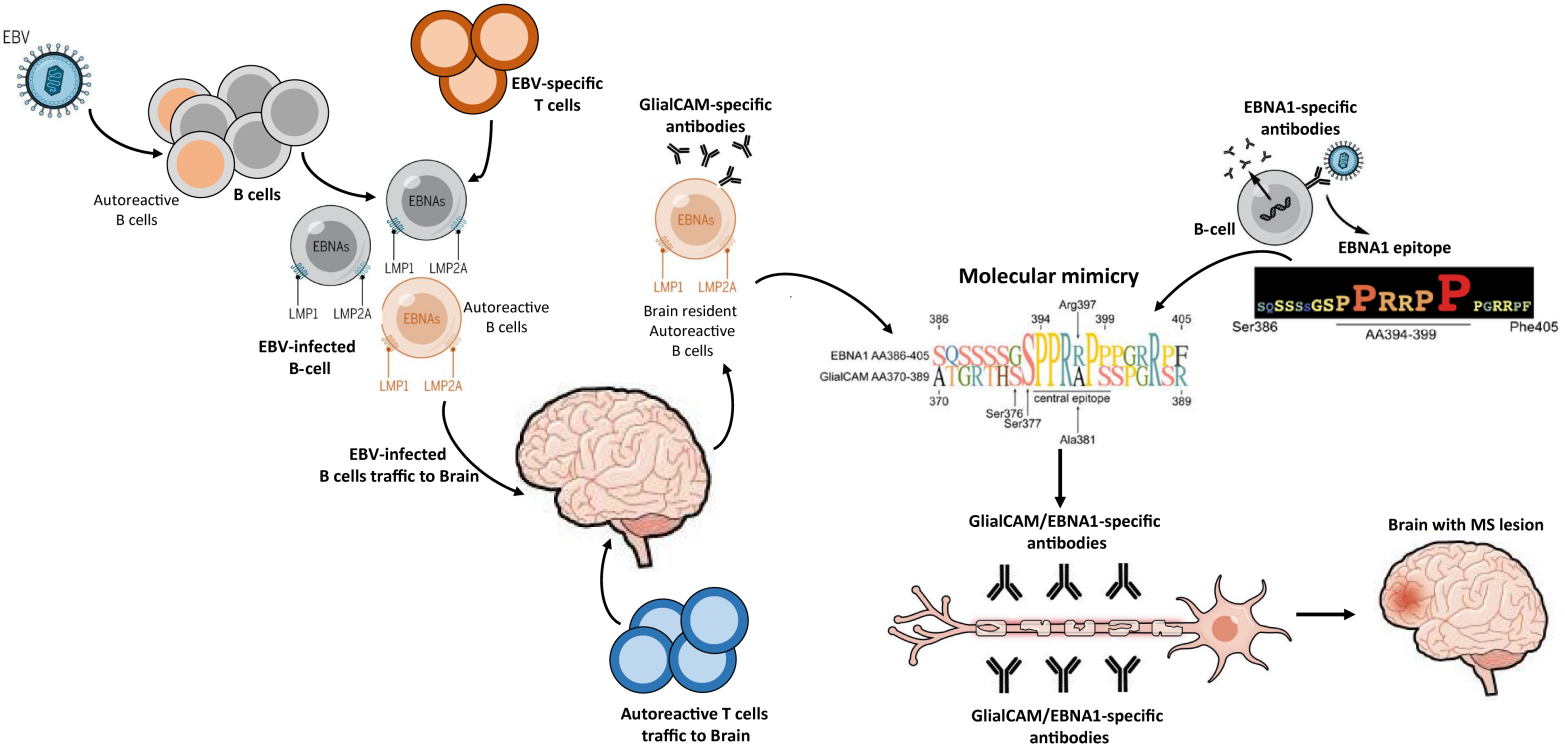
Primary acute Epstein–Barr virus (EBV) infection leading to inflammatory immune response and molecular mimicry recognising self‐antigens in central nervous system (CNS). Acute EBV infection can rescue autoreactive B cells which traffic to the brain leading to influx of T cells which result in inflammation in CNS. Autoreactive B cells secrete antibodies specific for EBV‐encoded EBNA1 protein, which cross‐reacts with CNS antigens (e.g. GlialCAM). These antibodies contribute to the pathogenesis of multiple sclerosis (MS).

Another important aspect of the link between EBV and MS is the molecular mimicry with cross‐reactive autoantibodies recognising EBV proteins and self‐antigens (Figure 2).^2^ Of particular interest is the elevated levels of EBNA1‐specific antibodies in their serum and cerebrospinal fluid of MS patients.^57^, ^58^, ^59^ These elevated levels of EBNA1‐specific antibodies have been recognised as a biomarker for an increased risk of MS.^60^ Furthermore, T cells specific for EBNA1 antigens have been shown to recognise myelin basic protein, anoctamin 2, α‐crystallin B chain and more recently glial cell adhesion molecule (GlialCAM).^2^, ^61^, ^62^, ^63^ Other EBV‐specific T cells directed to viral lytic antigens including BHRF1 and BPLF1 cross‐react with self‐antigen RASGRP2 which is expressed in the brain.^64^ While the precise physiological relevance of these cross‐reactive antibodies and T cells in the pathogenesis of MS remains unresolved, it will be important to carefully consider the potential risk of targeting EBV‐encoded proteins while designing virus‐specific immune‐based therapeutic strategies.

## Augmenting immune control of EBV in MS patients using T‐cell immunotherapy

Immune modifying approaches have long been used to treat patients with MS. A brief summary of these therapies and their mechanism of action is outlined in Table 1. Knowledge gained on the EBV‐associated antigen profile in the autopsied brains of MS patients^49^, ^54^ and our understanding of the immune evasion mechanisms of the latency II proteins provides the basis for the hypothesis that the inefficient control of EBV‐infected B cells expressing EBNA1 and LMP1 &2 contributes to the pathogenesis of MS.^65^, ^66^ Consequentially, a strategy to augment EBNA1 and LMP1&2‐specific T‐cell immunity could have the potential to eliminate EBV‐infected cells associated with MS pathogenesis. Previous observations, particularly in EBV‐associated PTLD, have used adoptive T‐cell therapy to eliminate transformed B cells, which has been highly effective at controlling disease.^67^ EBNA1‐ and LMP1&2‐specific T cells are subdominant in the periphery of EBV‐exposed individuals and standard approaches to generate T‐cell therapies for PTLD bias towards the expansion of EBNA3‐6‐specific T cells. To overcome this limitation, we developed the AdE1‐LMPpoly vector for latency II‐associated diseases.^68^ The AdE1‐LMPpoly was designed to limit the immune evasion strategies of EBNA1 and LMP1 & 2 and encodes an EBNA1 gene without the sequence encoding the GAr, fused to a polypeptide of 16 MHC class I‐restricted epitopes derived from LMP1 & 2. Initially designed as a potential vaccine for diseases associated with an EBV latency II profile, it has proven very efficient at preferentially expanding EBNA1 and LMP1 & 2‐specific CD8^+^ T cells from healthy volunteers, cancer patients and MS patients and has been used in multiple clinical studies.^9^, ^30^, ^68^, ^69^ Similar T‐cell therapy and vaccine approaches have been developed by other groups and designed to target the latency II proteins.^42^, ^70^ Small molecule inhibitors are also under development that specifically target these proteins.^71^, ^72^

**Table 1:** Immune modifying therapeutic strategies for the treatment of MS

Therapeutic agent	Drug compositions	Mechanism of action	References
Approved therapies			
β‐Interferon	IFN‐β‐1b, IFN‐β‐1a and pegylated IFN‐β‐1b	Alteration of dendritic cell function, expression of co‐stimulatory molecules and cytokines Limit CD4^+^ and CD8^+^ T‐cell effector function Enhance the development of CD4^+^ Treg cells Dampening B‐cell responses by inducing apoptosis of pathogenic B cells	77, 78, 79, 80
Glatiramer acetate	Composed of a mixture of synthetic polypeptides consisting of four amino acids found in myelin basic protein: glutamic acid, lysine, alanine and tyrosine	Binds to MHC class II on antigen‐presenting cells and blocks loading of autoreactive peptides Skewing of autoreactive pathogenic T‐cell response to regulatory function	80, 81
Dimethyl fumarate (DMF)	Bioequivalent diroximel fumarate	Changes differentiation of various effector CD4^+^ T cells (specifically a shift from T helper Th1/Th17 to Th2 profile) Expansion of Tregs Inhibition of B and NK cell responses	80, 82
Sphingosine‐1‐phosphate receptor modulators	Fingolimod, siponimod, ozanimod and ponesimod	Promote lymphocyte egress from secondary lymphoid tissues through S1P receptor 1 signalling Inhibits trafficking of lymphocytes from lymph nodes to central nervous system, thus preventing relapses	80, 83, 84, 85
Mitoxantrone	Synthetic anthracenedione derivate	Promote death of proliferating T and B cells by disrupting DNA synthesis and DNA repair	80, 86
Cladribine	Purine analogue	DNA strand breakage and apoptosis of proliferating and activated lymphocytes	80
Teriflunomide	Pyrimidine analogue	Inhibits *de novo* pyrimidine synthesis by blocking the enzyme dihydroorotate dehydrogenase leading to the apoptosis of proliferating T and B cells	80
Natalizumab	Monoclonal antibody specific for α4‐integrin	Prevents interactions between vascular cell adhesion molecule‐1 and α4β1‐integrin thus preventing the transmigration of autoreactive T cells into the CNS	80, 87
Ocrelizumab	Monoclonal antibody specific for CD20	Depletion of immature and mature B cells	80
Alemtuzumab	Monoclonal antibody specific for CD52	Antibody‐dependent, cell‐mediated cytolysis and complement‐dependent cytolysis of activated T cells and B cells	80, 88
Therapies in clinical trials			
Simvastatin	HMG‐CoA reductase inhibitor	Altering dendritic cell function and maturation and reduction in MHC class II antigen presentation Reducing effector CD4^+^ T‐cell responses Prevent leukocyte migration across the blood–brain barrier	89
Minocycline	Tetracycline antibiotic	Reduction of microglial activation Blocks trafficking of leukocytes across blood–brain barrier by inhibiting matrix metalloproteinases	90
Ibudilast	Phosphodiesterase inhibitor	Blocks macrophage migration inhibitor factor Reduction of IL‐1β, TNF‐a and IL‐6 production TLR4 antagonist	91
Dendritic cell vaccination	Autologous dendritic cells sensitised with myelin peptide and tolerised with 1α,25 dihydroxyvitamin D3	Antigen‐specific T‐cell tolerance	92, 93
NeuroVax	Includes three peptides (BV5S2, BV6S5 and BV13S1) emulsified in incomplete Freund's adjuvant	Induces FoxP3^+^ Tregs and IL‐10‐secreting T regulatory 1 (Tr1) cells	94
AHSCT	Autologous haematopoietic stem cell transplant	Eliminates autoreactive immune cells	95
Adoptive T‐cell therapy	Autologous EBV‐specific T‐cell therapy directed to EBNA1, LMP1 & LMP2	Killing of EBV‐infected B cells	8, 9
ATA188	Allogeneic ‘off‐the‐shelf’ EBV‐specific T‐cell therapy directed to EBNA1, LMP1 & LMP2	Killing of EBV‐infected B cells	96

EBV, Epstein–Barr virus; MS, multiple sclerosis.

The first MS patient treated with EBV‐specific T‐cell therapy was recruited under the Australian Therapeutic Goods Administration special access scheme.^8^ This patient received an autologous T‐cell therapy product that comprised T cells specific for EBNA1 and LMP1&2. Following the administration of four escalating doses of EBV‐specific T cells, there was evidence of symptomatic improvement, including a decrease in the number of brain lesions and intrathecal IgG. This case report was followed by an open‐label phase I study using autologous EBV‐specific T cells to treat five patients with primary progressive and five patients with secondary progressive MS.^9^ Patients received four escalating doses of AdE1‐LMPpoly generated EBV‐specific T cells. The therapy was well tolerated with no investigational product‐related severe adverse events. Seven of the ten patients treated with T cells showed evidence of symptomatic improvement that included reduced fatigue and in some cases an improvement in EDSS score. This symptomatic improvement was associated with higher EBV specificity in the cell therapy products and a strong polyfunctional profile, evidence of correlates of T‐cell potency with clinical improvement. Follow‐up analysis by our clinical collaborators has indicated that these symptomatic improvements are maintained in patients.^73^

**Figure 3:**
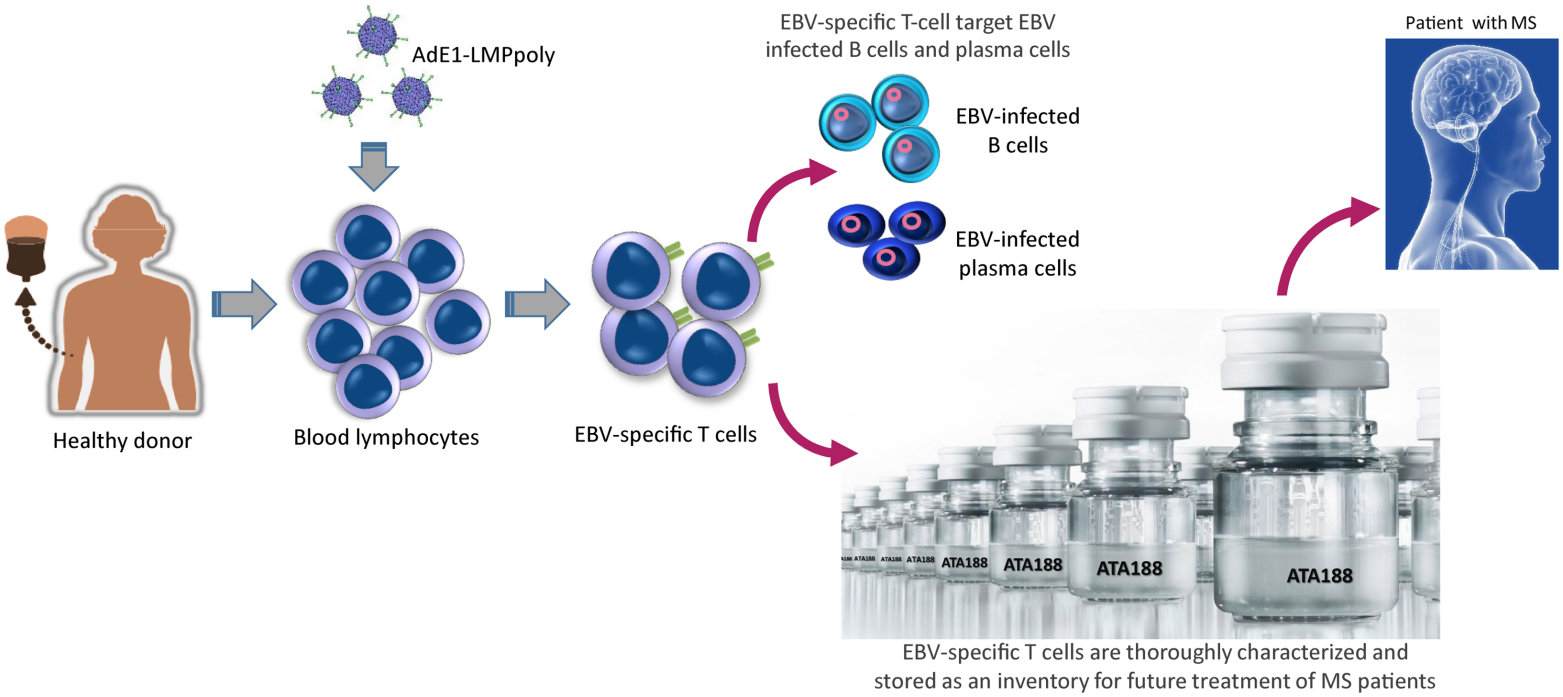
Overview of manufacturing of allogenic ‘off‐the‐shelf’ Epstein–Barr virus (EBV)‐specific T‐cell therapy for the treatment of multiple sclerosis (MS) patients. Peripheral blood mononuclear cells from healthy EBV seropositive individuals are stimulated with a recombinant replication‐deficient adenovirus encoding EBV antigens. These cells are then expanded for 14–17 days and stored as an inventory and matched with MS patients based on HLA typing. These T cells are specifically designed to target EBV‐infected B cells and plasma cells.

A limitation of our autologous study was the failure to generate EBV‐specific T cells from 3 of 13 patients recruited to the study and the low EBV specificity in others that may contribute to reduced efficacy. We have now developed an allogeneic off‐the‐shelf EBV‐specific T‐cell therapy which offers an alternative to autologous T‐cell therapy that uses healthy EBV seropositive volunteers as a source for cellular immunotherapy^74^ (Figure 3). The use of healthy volunteers provides the advantage of using predefining characteristics to allow the selection of optimal donors to improve the potency and consistency of the cell therapies. They also offer a more practical approach for the widespread use of T‐cell therapies by overcoming the limitation of individualised autologous therapies for each individual patient. Atara Biotherapeutics has now completed a Phase I open‐label study assessing the safety of ATA188, an off‐the‐shelf product generated using the AdE1‐LMPpoly vector, in individuals with progressive MS.^75^, ^76^ In this study, it has been reported that nine of the 24 MS patients treated with ATA188 T‐cell therapy achieved sustained disability improvement which was based on the improvement in Expanded Disability Status Scale (EDSS) scores or reduction in the time needed to walk 25 feet. Furthermore, 13 patients showed stable EDSS scores and four patients experienced confirmed disability progression. More importantly, patients treated with ATA188 T‐cell therapy showed no treatment‐related grade > 3 adverse events, dose‐limiting toxicities, cytokine release syndrome, graft vs host disease or infusion‐related reactions. Remarkably, after 4 years of follow‐up, five patients maintained their clinical improvements with medial duration of improvement of 27.5 months, while eight patients with stable EDSS score also remained stable for a median of 41.2 months. In addition to these clinical benefits, patients who demonstrated confirmed disability improvement also indicated evidence of increased magnetisation transfer ratios (MTR) on MRI scans, which is considered as a biomarker of myelin density. Atara has since initiated a Phase 2 randomised, double‐blind placebo‐controlled trial (EMBOLD) evaluating the efficacy and safety of ATA188 in people with progressive forms of MS.

## Conclusions

While the contribution of brain‐resident EBV‐transformed B cells to the pathogenesis of MS remains to be fully elucidated, our thorough understanding of the latent lifecycle of EBV, and the immune mechanisms that control persistent infection, has enabled the translation of an EBV‐specific cellular therapy initially developed to treat EBV‐associated malignancies into a promising treatment for patients with progressive MS. This adoptive immunotherapy is specifically designed to target EBV‐infected autoreactive B cells which may be contributing to the pathogenesis of MS. Clinical studies using both autologous and allogeneic T‐cell therapies have shown that this therapeutic strategy is safe with minimal side effects and clinical benefit observed in some patients is sustained for long‐term. In future, this T‐cell therapy may be complemented with a therapeutic vaccine which specifically enhances T‐cell immunity to EBV latent antigens, which are expressed in EBV‐infected B cells.

## Conflict of interest

CS and RK hold international patents on work discussed in this manuscript. This work has been licensed to ATARA Biotherapeutics. They consult to ATARA Biotherapeutics on their EBV T‐cell immunotherapy programme. RK is also appointed to the Scientific Advisory Board of Atara Biotherapeutics.

## Author contributions

**Corey Smith:** Writing – original draft; writing – review and editing. **Rajiv Khanna:** Writing – original draft; writing – review and editing.
